# Draft Genome Sequence of the Basidiomycetous Yeast-Like Fungus *Pseudozyma hubeiensis* SY62, Which Produces an Abundant Amount of the Biosurfactant Mannosylerythritol Lipids

**DOI:** 10.1128/genomeA.00409-13

**Published:** 2013-06-27

**Authors:** Masaaki Konishi, Yuji Hatada, Jun-ichi Horiuchi

**Affiliations:** Department of Biotechnology and Environmental Chemistry, Kitami Institute of Technology, Kitami-shi, Hokkaido, Japana; Department of Biogeoscience (Biogeos), Japan Agency of Marine-Earth Science and Technology (JAMSTEC), Yokosuka, Kanagawa, Japanb

## Abstract

The basidiomycetous yeast-like fungus *Pseudozyma hubeiensis* strain SY62 is capable of producing an abundant amount of the glycolipid biosurfactant mannosylerythritol lipids (MELs), which are a major component of monoacetylated MEL (MEL-C). To reveal the synthetic pathway of the MELs of strain SY62, we present the 18.44-Mb draft genome sequence.

## GENOME ANNOUNCEMENT

Mannosylerythritol lipids (MELs) are one type of glycolipid biosurfactant, which are produced by several species of basidiomycetous yeast-like fungi, including various species in the genera *Ustilago* and *Pseudozyma* (1). The molecules consist of 4-*O*-β-d-mannopyranosyl-meso-erythritol with fatty acid esters. Further, their reported functions are not only their excellent surface activities, but also their unique activities, including self-assembly, antitumor, and cell differentiation-inducing activities (2). The genome sequence of *Pseudozyma antarctica*, as a typical MEL producer, has reportedly been investigated recently (3). However, MELs that are produced by organisms within the genus *Psuedozyma* have a variety of molecular structures and functions (4). In particular, the acetylation and acylation of mannose moiety are assumed to play significant roles in the self-assembling behaviors of MELs (4).

*Pseudozyma hubeiensis* strain SY62 was isolated from the deep sea in Sagami Bay, Japan, as a fungus that mostly produces 4-*O*-[4′-*O*-acetyl-2′,3′-di-O-alka(e)noyl-β-d-mannopyranosyl]-d-erythritol (MEL-C) (5). Glycosyltransferase (Emt1), acyltransferases (Mac1 and Mac2), acetyltransferase (Mat1), and a major facilitator (Mmf1) are included in the MEL synthetic pathway in the related species *Ustilago maydis* (6) and *P. antarctica* (4). These species mostly produce a diacetylated component of MELs, 4-*O*-[4′,6′-*O*-diacetyl-2′,3′-di-*O*-alka(e)noyl-β-d-mannopyranosyl]-d-erythritol (MEL-A) (3). Here, we present the genome sequence of *P. hubeiensis* SY62 as a representative of MEL-C producers, and we compare its sequence to those of the other producers in order to discuss the differences in MEL synthetic metabolism and the functions of the genes.

Draft sequencing was performed by the Illumina HiSeq system with a total of 62,228,512 reads. The sequence reads from the paired-end library (400 bp) were initially assembled into 160 contigs and 74 scaffolds using Augustus v1.2.08. The contigs include a total genome size of 18,442,938 bp, and the G+C content is 56.5%. The estimated genome size and G+C content of SY62 correspond approximately to those of strains *U. maydis* 521 (6) and *P. antarctica* T-34 (4).

The coding regions and their functions were predicted using MetaGeneAnnotator 1.0 and NCBI BLAST 2.2.18. The rRNA and tRNA genes were found using RNAmmer and tRNAscan, respectively. The draft genome is composed of 7,523 putative coding genes or open reading frames (ORFs), 26 rRNA genes, and 121 tRNA genes. The genes of the cluster for MEL synthesis, *emt1*, *mac1*, *mac2*, *mmf1*, and *mat1*, were observed in the genome of *P. hubeiensis* SY62. The translated amino acid sequences of *emt1*, *mac1*, *mac2*, *mmf1*, and *mat1* in the SY62 genome show identities of 79.7, 71.6, 62.1, 82.2, and 57.8% to the corresponding genes in *U. maydis* and identities of 77.1, 61.2, 50.7, 77.6, and 54.5% to the corresponding genes in *P. antarctica*, which is mostly a 4-*O-*[4′,6′-di-*O*-diacetyl-2′,3′-alkanoyl[-β-d-mannopyranosyl]-meso-erythritol (MEL-A)-producing species. The low identity of Mat1 between *P. hubeiensis* and the other MEL-producing fungi seemed to be associated with the differences of the specificity of acetyltransferase and the resulting difference of the acetylation of major products. We are investigating the functions of the genes by using deletion mutants. These mutants will be also useful for the selective production of metabolic intermediates.

### Nucleotide sequence accession numbers.

This Whole-Genome shotgun project has been deposited at DDBJ/EMBL/GenBank under accession no. BAOW01000001 to BAOW01000160 (as 160 entries) and DF238764 to DF238837 (as 74 scafffolds).
